# Study of V_2_CT_*x*_-MXene Based Immunosensor for Sensitive Label-Free Impedimetric Detection of SARS-CoV-2 Spike Protein

**DOI:** 10.1021/acsami.4c04567

**Published:** 2024-05-30

**Authors:** Nikola Tasić, Ivan Konjević, Alnilan Lobato, Dino Metarapi, Matjaž Finšgar, Filipa M. Oliveira, Zděnek Sofer, Rui Gusmão, Xueji Zhang, Samo B. Hočevar

**Affiliations:** † Department of Analytical Chemistry, National Institute of Chemistry, Hajdrihova ulica 19, 1000 Ljubljana, Slovenia; ‡ Faculty of Chemistry and Chemical Technology, University of Ljubljana, Večna pot 113, 1000 Ljubljana, Slovenia; § International Postgraduate School Jožef Štefan, Jamova 39, 1000 Ljubljana, Slovenia; ∥ Faculty of Chemistry and Chemical Engineering, University of Maribor, Smetanova ulica 17, 2000 Maribor, Slovenia; ⊥ Department of Inorganic Chemistry, University of Chemistry and Technology Prague, Technická 5, 166 28 Praha 6-Dejvice, Czech Republic; # School of Biomedical Engineering, Shenzhen University Health Science Center, 3688 Nanhai Road, Nanshan District, Shenzhen 518054, Guangdong P.R. China

**Keywords:** V_2_CT_*x*_ MXene, SARS-CoV-2, spike protein, immunosensor, electrochemical, impedimetric

## Abstract

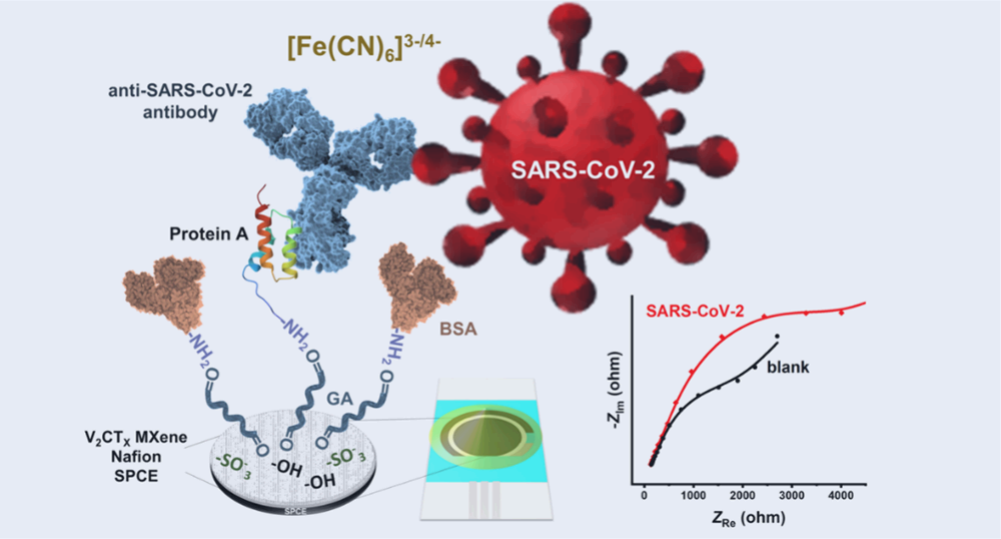

Rapid and reliable immunosensing is undoubtedly one of the priorities in the efficient management and combat against a pandemic, as society has experienced with the SARS-CoV-2 outbreak; simple and cost-effective sensing strategies are at the forefront of these efforts. In this regard, 2D-layered MXenes hold great potential for electrochemical biosensing due to their attractive physicochemical properties. Herein, we present a V_2_CT*_x_* MXene-based sensing layer as an integral part of a label-free immunosensor for sensitive and selective detection of the SARS-CoV-2 spike protein. The sensor was fabricated on a supporting screen-printed carbon electrode using Nafion as an immobilizing agent for MXene and glutaraldehyde, the latter enabling effective binding of protein A for further site-oriented immobilization of anti-SARS-CoV-2 antibodies. A thorough structural analysis of the sensor architecture was carried out, and several key parameters affecting the fabrication and analytical performance of the immunosensor were investigated and optimized. The immunosensor showed excellent electroanalytical performance in combination with an impedimetric approach and exhibited a low detection limit of only 45 fM SARS-CoV-2 spike protein. Its practical applicability was successfully demonstrated by measuring the spike protein in a spiked artificial nasopharyngeal fluid sample.

## 1 Introduction

The world has suffered a huge socioeconomic disaster in the context of the SARS-CoV-2 pandemic. With the emergence of potentially more transmissible variants and their possible resistance to commercially available vaccines, societies undoubtedly face the challenge of ensuring adequate prevention and treatment strategies.^1,2^ Consequently, the endeavors of the research teams to fabricate and validate viable alternatives to the commercially available RT-PCR (reverse transcription polymerase chain reaction) or widely exploited serological tests are still underway.^3^

It is known that electrochemical immunosensors are capable of detecting trace amounts of biological molecules in a highly selective manner, with a rapid response, by making use of miniaturized devices that nominates them for point-of-need applications.^4−7^ The existing literature on electrochemical detection of SARS-CoV-2 has already been reviewed elsewhere, suggesting that significant progress in this field has been made since the pandemic outbreak.^8,9^

The etching process of precursor MAX phases selectively removes the ‘A’ element layers (usually Al), exposing the MXene layers, which are laminated sheets of transition metal carbides, nitrides, or carbonitrides.^10^ MXenes are state-of-the-art 2D-layered nanostructures characterized by high electrical conductivity in both the in-plane and out-of-plane directions. Their large surface-to-volume ratio, high adsorption capacity, and thermal stability make them perfect candidates for smart sensing applications.^11^ These applications include the voltammetric detection of gaseous H_2_O_2_,^12^ humidity sensing,^13^ trace metals analysis,^14^ chemiresistive sensing,^15^ and piezoresistive sensors.^16^ However, as far as the development of sensing devices for SARS-CoV-2 is concerned, reports on MXenes are still very limited and restricted to biosensors based on field-effect transistors (FET),^17^ chemiresistive biosensors based on DNA-functionalized Ti_3_C_2_T_*x*_,^18^ luminescent nanoprobes,^19^ and electrochemiluminescent sensors.^20^

On the other hand, V_2_CT_*x*_ MXene has mainly been used in energy storage applications due to the spontaneous intercalation of various cations between its layers.^21^ Its potential in gas sensing has been demonstrated, owing to the surface oxygen functional groups present on the surface of V_2_CT_*x*_ nanoflakes and rich surface chemistry.^22^ Only recently it has been introduced into biosensing as conductive support for the in situ formation of spindle-shaped gold nanoparticles, serving as a metal tag for the antibodies and consequent detection of interleukin 6 from breast cancer cells.^23^

In this work, we developed an impedimetric immunosensor for SARS-CoV-2 spike protein based on V_2_CT_*x*_ MXene nanomaterial. We used a protocol for the chemical binding of anti-SARS-CoV-2 antibodies with glutaraldehyde (GA) as a cross-linker and protein A, facilitating the subsequent site-oriented binding of the antibody. Several studies were carried out encompassing the modification protocols and critical operational key parameters. Finally, we demonstrated a sensitive and selective detection of spike protein in PBS and nasopharyngeal samples within the time frame of ca. 15 min, making a step forward toward true point-of-need applications.

## 2 Methods

### 2.1 Chemicals

All commercial reagents were of analytical grade and were used without further purification. All aqueous solutions were prepared using ultrapure water with a resistivity of 18.2 MΩ cm at 298 K (Milli-Q, Millipore, Corp., Marlborough, MA). Potassium hexacyanoferrate(III) (K_3_Fe(CN)_6_), Nafion perfluorinated resin solution (5% in a mixture of lower aliphatic alcohols and water), and glutaraldehyde (GA) solution (25% in water) were purchased from Sigma-Aldrich. Sodium azide (NaN_3_) and bovine serum albumin (BSA) were purchased from Fluka. Potassium hexacyanoferrate(II) trihydrate (K_4_Fe(CN)_6_·3H_2_O) was purchased from Riedel de Haen. Potassium chloride (KCl), sodium chloride (NaCl), sodium hydrogen phosphate dihydrate (Na_2_HPO_4_·2H_2_O), and potassium dihydrogen phosphate (KH_2_PO_4_) were purchased from Merck. Absolute ethanol (C_2_H_5_OH) was purchased from Carlo Erba. Protein A (ab71456) and human monoclonal anti-SARS-CoV-2 spike glycoprotein S1 antibodies (ab286179) were purchased from Abcam. SARS-CoV-2 spike protein (S1 subunit, nCoV-P001) and nucleocapsid protein (protein N, nCoV-P003) were purchased from Bio Bench. HCoV-OC43 spike S1 protein (40607-V08H1), HCoV-HKU1 spike S1 protein (40021-V08H), HCoV-NL63 spike S1 protein (40600-V08H), and HCoV-229E spike protein (40605-V08B), and MERS spike protein (40069-V08B) were all purchased from Sino Biological Europe GmbH, Eschborn, Germany. The artificial saliva was purchased from BioChemazone. The chemicals used in the MAX phase etching were all from Sigma-Aldrich, except when specified otherwise.

### 2.2 Preparation of Buffer Solutions

Phosphate-buffered saline (PBS, pH = 7.4) was prepared by dissolving 8.00 g of NaCl, 200.0 mg of KCl, 1.81 g of Na_2_HPO_4_·2H_2_O, and 240.0 mg of KH_2_PO_4_ in 1.0 L of purified H_2_O. When the PBS was used to dilute proteins, 0.001% NaN_3_ was added to the solution as a preservative.

### 2.3 Synthesis and Characterization of V_2_CT*_x_* MXene

The synthesis of multilayered V_2_CT_*x*_ MXene powder involved selectively removing Al from V_2_AlC powder. Initially, 5.0 g of V_2_AlC MAX phase material sourced from Jinzhou Haixin Metal Materials in China was gradually added to a solution of 250.0 mL of 40.0% hydrofluoric acid (HF). This mixture was continuously stirred for 48 h within a 500 mL capacity Teflon-lined stainless-steel autoclave. Subsequently, the sample was stirred for an additional 48 h in hydrochloric acid (HCl) to a concentration of 6 mol L^–1^. For the final etching step, the sample was immersed in a mixture of lithium fluoride (LiF, ≥98.5 wt %), 6 mol L^–1^ HCl, and deionized water for 72 h. Following the etching process, the sediment underwent multiple washes with deionized water until the pH of the supernatant reached approximately 6, followed by a wash with absolute ethanol. Finally, the obtained powders were dried in a vacuum for 24 h at a temperature of 60 °C.

### 2.4 Modification of the Supporting Electrode and Sensor Preparation

**Figure 1:**
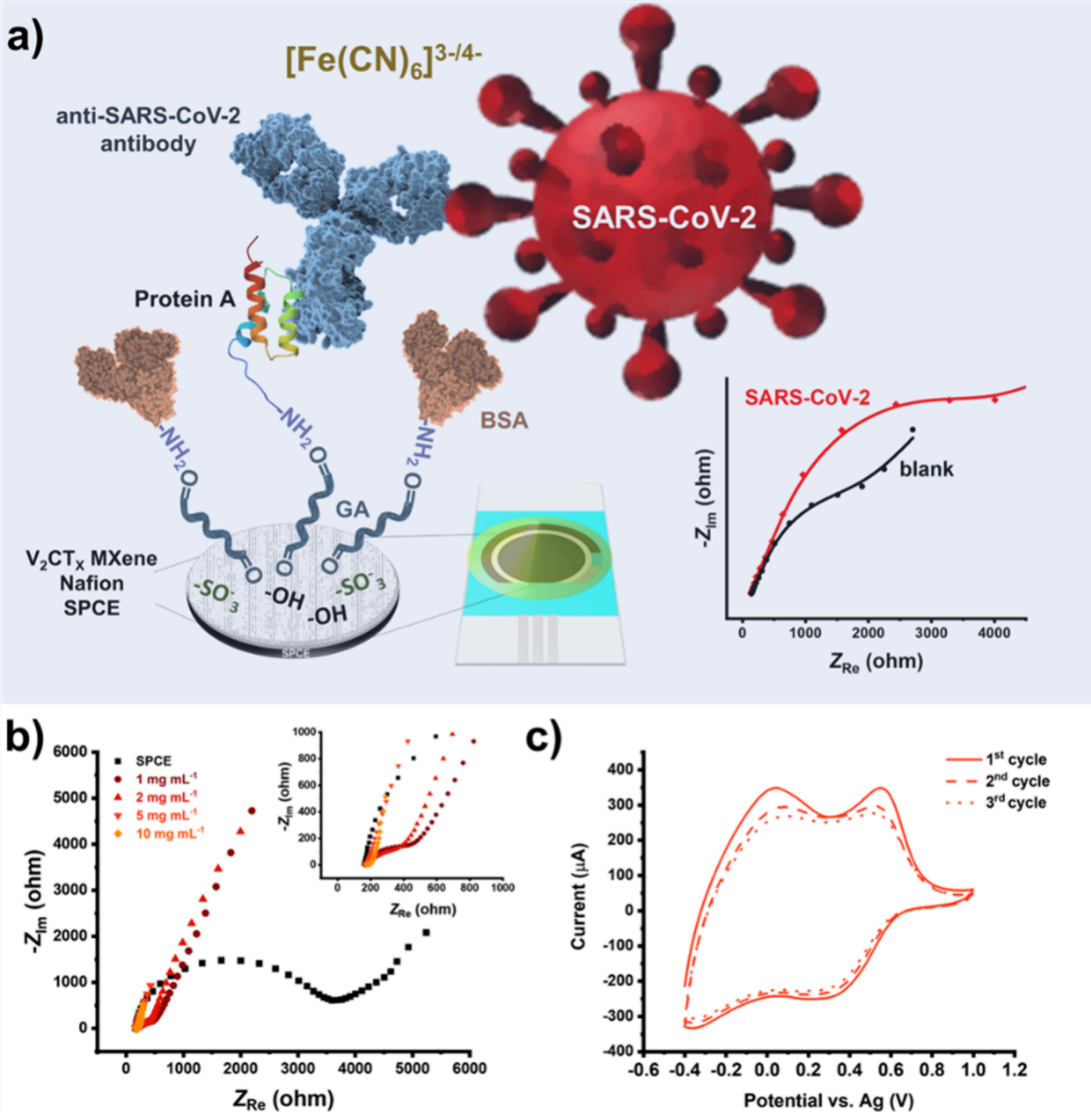
(a) A detailed scheme of the immunosensor architecture and its detection principle, (b) Nyquist spectra of the V_2_CT_*x*_ MXene-modified SPCEs using 1.0 mM [Fe(CN)_6_]^3–/4–^ + 0.1 M L^–1^ KCl as a redox probe. The inset shows a magnified high-frequency region of the spectra, and (c) CVs of SPCE modified with 2 mg mL^–1^ V_2_CT_*x*_ MXene in 0.1 M KCl.

The working electrode of the screen-printed carbon electrode (SPCE, DropSens, DRP-110, Metrohm, Herisau, Switzerland, diameter of 4 mm) was first modified with V_2_CT_*x*_ MXene by drop casting 10 μL of its 2 mg mL^–1^ aqueous solution and left at ca. 35 °C to dry in the closed and light-protected dish. Prior to this, the drop-casting solution was sonicated for 15 min (Elma Transsonic Digital S ultrasound bath, frequency of 40 kHz, 100% applied sonication power at 22–30 °C). In the next step, the working electrode was covered with a Nafion protective/immobilization layer by drop casting 2.2 μL of the 0.0025% Nafion solution in ethanol. After drying at room temperature (23 °C, ca. 1 h), the working electrode was modified with 10 μL of 2.5% GA solution in PBS (pH 7.4) for 2 h at 4 °C, in a light-protected dish. Then the electrode was rinsed carefully by immersing it in purified water for ca. 5 s and dried for ca. 10 s with nitrogen at 0.2 bar. In the following step, the electrode was incubated using 10 μL of 2 μg mL^–1^ protein A solution in PBS (pH 7.4) for 1 h, at room temperature, and then rinsed and dried again in the same way as after the addition of GA. Afterward, the working electrode was incubated with 10 μL of 2 μg mL^–1^ anti-SARS-CoV-2 antibody solution in PBS (pH 7.4) for 30 min. The electrodes were then kept at 4 °C overnight to reach the conformational stability of proteins. The remaining nonspecific binding sites were covered with BSA by exposing the working electrode of the SPCE to 10 μL of 10 μg mL^–1^ BSA solution in PBS for 30 min, at room temperature. Finally, the electrodes were rinsed with purified water and dried with nitrogen to eliminate liquid residues on the surface and were ready for use. When not used, the immunosensors were stored in a closed vessel in the refrigerator at 4 °C. A detailed scheme of the immunosensor architecture and its detection principle is shown in Figure 1a.

### 2.5 Electrochemical Measurements

All electrochemical measurements, i.e., cyclic voltammetric (CV) and electrochemical impedance spectroscopic (EIS), were performed using a portable PalmSens 4 potentiostat/galvanostat controlled by the PSTrace 5.9 software (PalmSens BV, Netherlands). Electrochemical impedance spectroscopy (EIS, Nyquist plots) measurements were performed in the frequency range of 10^4^–10^–1^ Hz at the potential of +0.14 V vs integrated silver quasi-reference electrode at the SPCE, with an amplitude of 5 mV, using 1.0 mM [Fe(CN)_6_]^3–/4–^ in 0.1 M KCl as a redox probe. As the analytical signal, the difference between the *R*_ct_ value extracted from the measurement of the incubated sensor (*R*_inc_) and the *R*_ct_ value of the nonincubated sensor (*R*_blank_), i.e., *R*_inc_ – *R*_blank_, was used. These values were obtained by fitting the raw impedance data using the equivalent electrical circuit *R*_S_(*R*_MXene_*Q*_MXene_)([*R*_ct_*Z*_W_]*C*_dl_). Herein, *R*_S_ corresponds to the solution resistance, *C*_dl_ is the double-layer capacitance, *R*_ct_ is the charge transfer resistance of the sensing layer/electrolyte interface, and *Z*_W_ is the Warburg element that models the diffusion phenomenon; *R*_MXene_ and *Q*_MXene_ correspond to the resistance and the constant phase element of the nonideal capacitance of the underlying V_2_CT_*x*_ MXene layer, respectively.

### 2.6 Characterizations

The X-ray photoelectron spectroscopy (XPS) measurements were performed using a Supra+ device (Kratos, Manchester, UK) and an Al K_α_ excitation source. The binding energy (BE) scale was corrected based on the C–C/C–H peak in the C 1s spectra. High-resolution spectra were acquired at a 90° takeoff angle. Sputtering was performed with a 5 keV Ar_2000_^+^ gas cluster ion beam rastering over 2 by 2 mm. During the depth profiling, spectra were acquired in the center of the sputter crater on a 110-μm spot size (in diameter) at the pass energy of 40 eV. Spectra acquisition and processing were performed using ESCApe 1.5 software (Kratos).

Time-of-flight secondary ion mass spectrometry (ToF-SIMS) measurements were conducted using a M6 device (IONTOF, Munster, Germany) and a 30 keV Bi^+^ primary beam (target current of 1.0 pA). Spectra acquisition and processing were performed with SurfaceLab 7.3 software (IONTOF). 3D depth profiles were obtained using 2.5 keV Ar_1900_^+^ gas cluster ion beam rastering over 500 by 500 μm and the analysis of an area of 300 by 300 μm in the middle of the sputter crater. Spectra calibration was performed with the signals at known *m*/*z*.

The morphology of the exfoliated materials was observed by Tescan Maia 3 with a field emission gun (Tescan, Czech Republic). The measurements were performed at an acceleration voltage of 20 keV. An SDD detector (X-MaxN 80 TS) from Oxford Instruments (England) was used to obtain the elemental maps and EDX spectra. The materials were sonicated in an aqueous solution for 20 min in an ultrasonic bath (*T* < 30 °C, 37 Hz, 100%) to guarantee a dispersion of the MXene and drop-cast on a TEM grid (Cu; 200 mesh; Formvar/carbon). The drop-cast samples were dried under a vacuum. Field-emission scanning electron microscopy (FE-SEM) images and the EDX analysis of V_2_CT_*x*_ MXene deposited onto the SPCEs were obtained with a high-resolution scanning electron microscope (Carl Zeiss SUPRA 35 VP FE-SEM) equipped with an energy-dispersive X-ray spectrometer (Oxford Instruments Inca 400 EDX).

## 3 Results and Discussion

### 3.1 Characterization of V_2_CT_*x*_ MXene Layer

MXenes were introduced on the electrode surface by a drop-casting technique. It has been shown that a relatively small quantity of (nano)particles needs to be dispersed over the electrode surface to harvest the electrocatalytic potential of MXenes;^24^ thus, the concentration of the MXene suspensions in the initial experiments was in the range of 1–10 mg mL^–1^, corresponding to the final mass concentrations of 80–800 μg cm^–2^ in the deposited layer. In addition to a V_2_CT_*x*_ MXene layer, we also introduced a highly diluted Nafion coating (0.0025% in absolute ethanol) to immobilize the MXene particles at the electrode surface. The surface of the modified electrodes was first investigated using 3D interferometry, and the corresponding images are presented in Figure S1a–c. With MATLAB-compiled software, we calculated the average thickness of the V_2_CT_*x*_ MXene coatings using the prerecorded interferograms of the nonmodified and modified SPCEs as the source data. All data sets consisted of approximately 4600 imaging points per circular electrode area. The highest average thickness of the modification coating was observed when using 10 μL of 10 mg mL^–1^ MXene, i.e., ca. 1.5 μm in height, and significantly lower thicknesses for modifications with more diluted MXene solutions (see Figure S1a). The fluctuation of the thickness within the same sample, commonly present in drop-cast films, was elucidated by observing randomly chosen line sections through the approximate geometrical center of the modified electrode (see Figure S1c). Notably, the same trends in all samples were observed regardless of the chosen line section.

The initial electrochemical study of the V_2_CT_*x*_ MXene deposits was conducted by EIS analysis in the presence of 1.0 mM [Fe(CN)_6_]^3–/4–^ in 0.1 M KCl, and the corresponding Nyquist spectra are shown in Figure 1b. The spectra considerably changed by introducing the modification coatings using 1 and 2 mg mL^–1^ V_2_CT_*x*_ MXene in the drop-casting solutions compared to a nonmodified SPCE that exhibited a relatively high charge transfer resistance. The EIS spectra changed further with the increasing concentration of the MXene to 5 and 10 mg mL^–1^, showing a predominant contribution of the diffusion of redox species. When using the last two concentrations of V_2_CT_*x*_ MXene, a somewhat higher degree of irreproducibility for the impedimetric parameters was observed, which was not the case at lower concentrations of 1 and 2 mg mL^–1^ V_2_CT_*x*_ MXene (results not shown). This lack of reproducibility was manifested in larger standard deviations of EIS-fitted parameters (>25%) across replicate measurements. Additionally, the introduction of the highest examined MXene concentration of 10 mg mL^–1^ resulted in a certain extent (although low) of undesired material leaching. For this reason, we have decided to use electrodes modified with 2 mg mL^–1^ solution of V_2_CT_*x*_ MXene in all further studies. In addition, the electrodes modified with 2 mg mL^–1^ solution of V_2_CT_*x*_ MXene were characterized by CV, and the obtained voltammograms are presented in Figure 1c. Similarly to already investigated titanium MXene, i.e., Ti_3_C_2_T_*x*_, the V_2_CT_*x*_ MXene is also susceptible to electrooxidation, in this case to the stepwise electrooxidation in the anodic region at ca. +0.1 and +0.6 V, with a decrease and merging of the related peaks with further voltammetric cycles, implying the presence of metal oxide;^25^ likewise, a weak reduction signal at ca. +0.3 V still persists after subsequent scans. The matter of the innate MXene oxidation was already addressed in the case of Ti_3_C_2_T_*x*_,^26,27^ suggesting that the oxidation results from the anodization of metallic atoms at the defect sites and edges, and the extent of this electrochemical instability is mainly governed by pH of the electrolyte and thickness of the MXene flakes. Although most of the vanadium sites are likely to remain intact by the electrochemical process and do not transform into oxide, from the application perspective, this instability might be a favorable intrinsic property of the MXene-based materials. For example, the electrochemically induced coexistence of different vanadium valence states was successfully implemented for high-performing Zn-ion batteries.^28^ More specifically, a nanoscale vanadium oxide (VO_*x*_) coating effectively served for multielectron reactions while the sublayers of V_2_CT_*x*_ remained structurally intact and formed “so-called” nanochannels with intrinsically high conductivity, creating an overall synergy between the existing structural features. We also characterized the SPCE surfaces modified with the optimized amount of V_2_CT_*x*_ MXene, i.e., 2 mg mL^–1^, after sonication of as-synthesized material, and the corresponding images are shown in Figures S1b, S2a, and S2b. A 15 min sonication of the modification material resulted in almost complete delamination of the V_2_CT_*x*_ MXene particles, assuring uniform coverage of the electrode surface with the predominant existence of MXene-originating flakes and irregular submicrometer particles.

**Figure 2:**
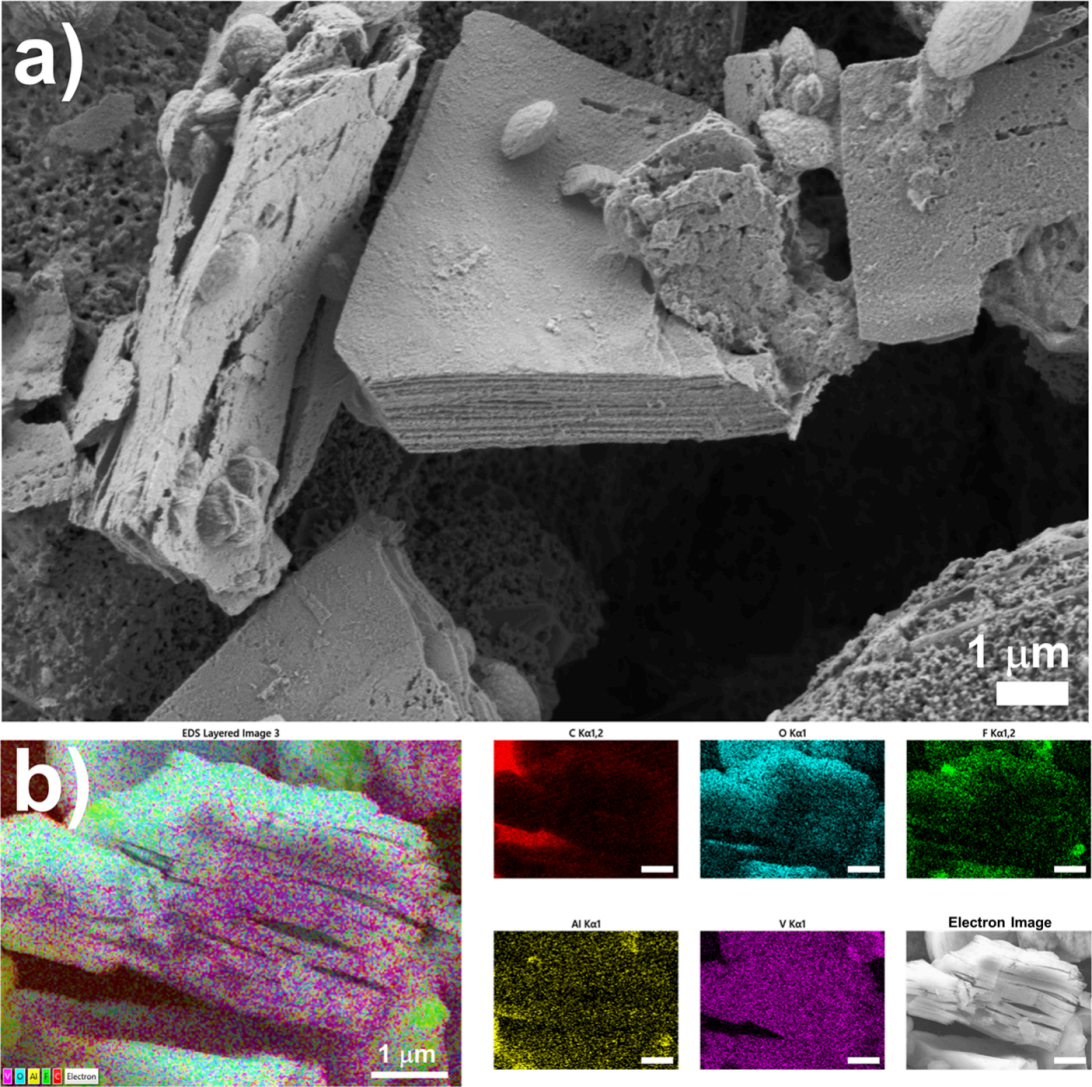
(a) FE-SEM analysis of the synthesized V_2_CT_*x*_ MXene and (b) EDX analysis of an accordion-like V_2_CT*_x_* MXene moiety.

In addition, we performed a morphology analysis of the synthesized V_2_CT_*x*_ MXene (Figure 2a). The etching process during MXene synthesis caused the removal of metallic interlayers from the precursor V_2_AlC MAX phase, resulting in an irregular, accordion-like morphology of the multilayered V_2_CT_*x*_ moieties.

As previously reported, the etching process is the most delicate step in MXene synthesis, where the removal of metallic interlayers can lead to the presence of different MXene structures, including MXene clay, intercalated MXene clay, and delaminated clay, accompanied by the degradation of MX layers to carbon and vanadium oxide.^29^ In other words, the phase structure of V_2_CT_*x*_ MXene, its structural integrity, and particle size distribution are strongly dependent on the synthesis of the precursor V_2_AlC MAX phase and the subsequent etching protocols. Under the given experimental conditions, we observed the coexistence of several features, which were confirmed by the presence of clay laminated moieties, delaminated clay, and irregular submicron oxide particles decorating the scattered MXene-based particles.

**Figure 3:**
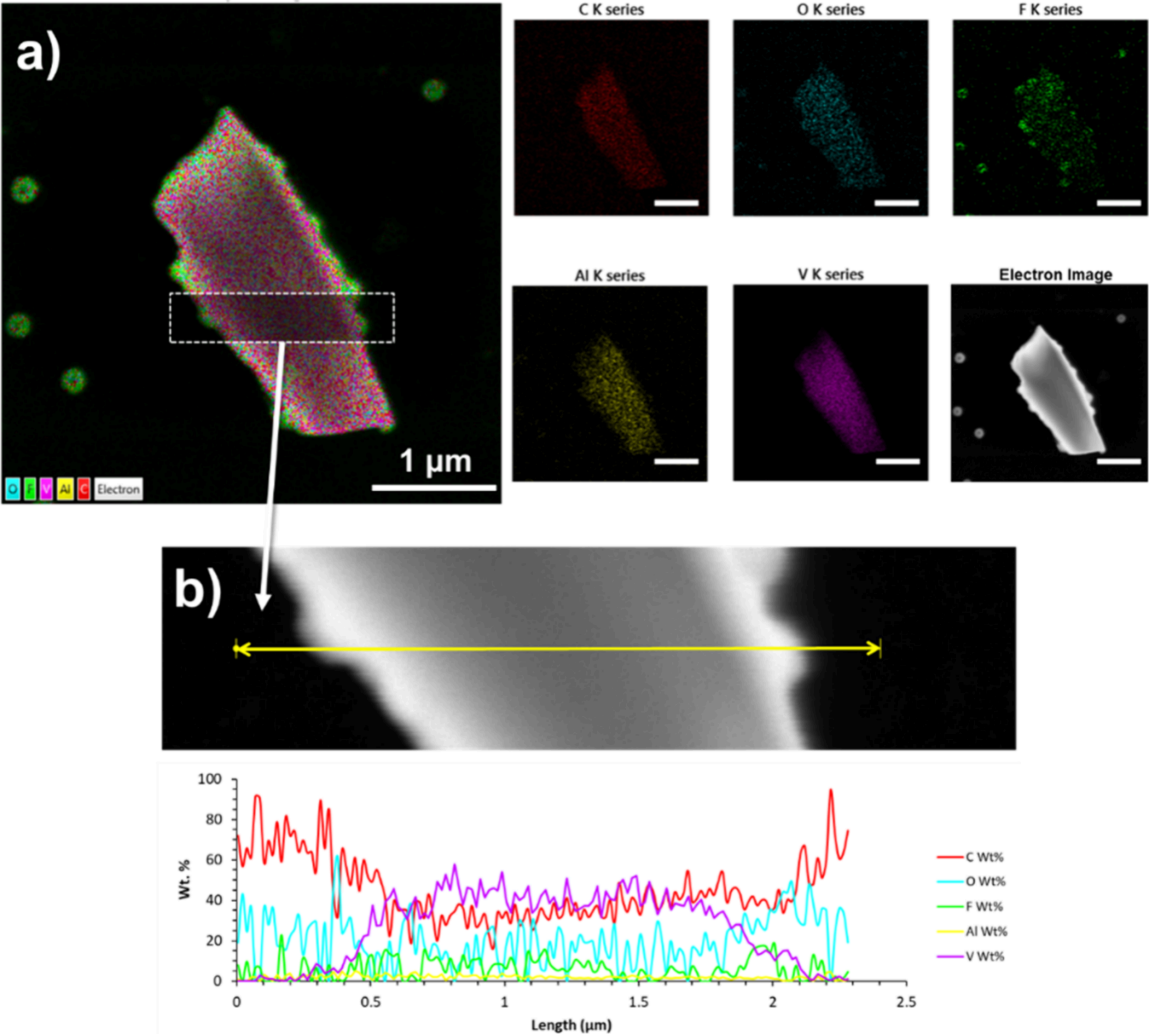
(a) Bright-field mode STEM/EDX analysis of the V_2_CT_*x*_ MXene flake with EDX layered image, respective map of elements and electron micrograph. (b) Detail of the area used for EDX 1D line scan analysis of the V_2_CT_*x*_ MXene flake and respective profile of detected elements (wt %). The scale bars represent 1 μm, and the length of the line scan is ca. 2.3 μm.

We also carried out an EDX analysis of the material shown in Figure 2b. The vanadium, oxygen, fluorine, and carbon were detected in all sampled areas of the material, corresponding mainly to MXene-based moieties and/or vanadium-based oxides. In addition, low amounts of aluminum impurities were detected, originating from the precursor MAX phase; however, their presence is not expected to significantly affect the functionality of the MXene-modified immunosensor. Oxygen was mainly present on the exposed surfaces of the as-synthesized material (see Figures S3a and S3b), and the corresponding elemental compositions are listed in Table S1. This is also supported by the STEM/EDX mapping of elements on individual micrometer scale flakes after the sonication (Figures 3, S4a, S4b). The midsection line scan analysis of the flake indicates that the distribution of O wt % is consistent across, while F wt % seems to be more abundant at the edges of the flakes. The C wt % is overestimated due to the evaporated carbon film characteristic of STEM grids. Moreover, V wt % has a noticeable increase within the region of the flake, up to 58%, while the Al wt % is kept under 7 wt % level. Oxygen can be attributed either to the terminal groups in the remaining MXene nanoflakes or to the presence of vanadium-based oxide nanoparticles. Finally, a significant amount of fluorine was observed, originating from the exfoliation agent, i.e., HF. Its presence is assigned to fluoride or oxyfluoride species and the terminal groups in MXene moieties.

### 3.2 Sensor Architecture Optimization Studies

GA is widely used as a cross-linking agent due to its high reactivity toward amino, hydroxyl, and carboxyl groups. In this study, GA was used to form a stable cross-link between the V_2_CT_*x*_ MXene/Nafion layer and protein A, the latter supporting the site-oriented immobilization of the anti-SARS-CoV-2 antibodies. The existing literature contains several articles in which proteins were directly adsorbed onto the composite layer of various nanoparticles with Nafion, such as carbon nanodots,^30,31^ carbon nanotubes,^32^ or multiwalled carbon nanotubes.^33^ However, the adsorption approach inevitably leads to protein denaturation, lower stability, and random alignment, resulting in sensors’ poorer analytical performance.^34^ Alternatively, the surface can be modified with different cross-linkers, e.g., MPA (3-mercaptopropionic acid) or EDC-NHS (carbodiimide 1-ethyl-3-(3-(dimethylamino)propyl)carbodiimide and *N*-hydroxysuccinimide),^35,36^ prior to the immobilization of antibodies, providing improved stability. Our approach exploits the reactivity of GA toward various end groups at the Nafion-coated V_2_CT_*x*_ MXene layer, such as sulfonate groups in Nafion and OH terminal groups in V_2_CT_*x*_, and its efficacy in immobilizing proteins.^37−39^ Nonetheless, GA protocols are designed empirically with respect to the binding protein and require optimization of pH, concentration, temperature, time, etc.^40^ Moreover, based on our experience, it is of utmost importance that the GA solutions are always freshly prepared prior to the modification step and that the subsequent reaction with the proteins is carried out instantly;^41^ otherwise, cross-linking can be compromised due to the intrinsic instability of GA. Our initial study (not shown) revealed that the modification with GA should be performed at 2–8 °C and under reduced light conditions to prevent its degradation. On the contrary, modifications performed under ambient and daylight conditions resulted in a nonreproducible electroanalytical behavior.

**Figure 4:**
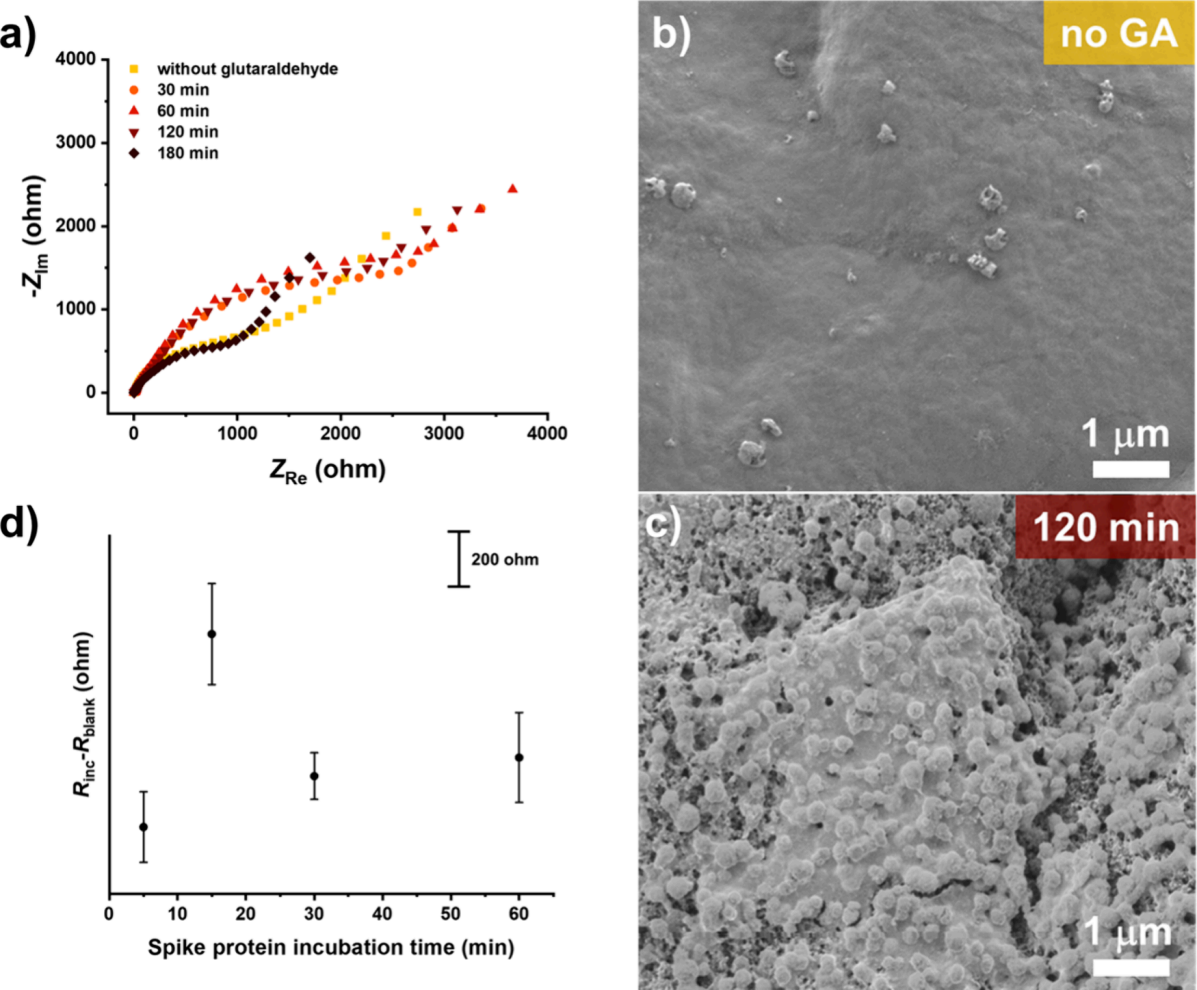
(a) Nyquist spectra of the V_2_CT*_x_*/Nafion SPCEs incubated for 1 h with 10 μg mL^–1^ protein A after modification with GA using different modification times, and without GA; surface morphology (FE-SEM) of (b) electrodes incubated with protein A without GA as a cross-linker, followed by incubation with protein A, and (c) electrodes modified with GA for 120 min, followed by incubation with protein A, and (d) effect of the SARS-CoV-2 spike protein incubation time upon the signal. The incubation drop volume was 10 μL, and the concentration of spike protein in PBS (pH = 7.4) was 10^–5^ μg mL^–1^.

The efficiency of GA cross-linking was investigated by EIS in the presence of 1.0 mM [Fe(CN)_6_]^3–/4–^ in 0.1 M KCl after the GA-modified electrode surface was incubated with 10 μg mL^–1^ protein A for 1 h. The experiments were carried out with different GA modification times ranging from 30 up to 180 min. For comparison, we also included the sample without GA as a cross-linker; Nyquist spectra show a depressed semicircle related to charge transfer resistance at the surfaces of the electrodes and diffusion tails related to the mass transfer of redox species (Figure 4a). The Nyquist spectrum of the electrode surface without GA exhibits a less resistive behavior than surfaces with the GA (up to 120 min), indicating that the direct adsorption approach without a cross-linker leads to lower coverage of the electrode surface with protein A. On the other hand, the extended modification time of 3 h for GA exhibited lower resistance, indicating degradation of GA in water-based solutions.^42^ In addition, we examined protein A-modified SPCE by FE-SEM and observed abundant cross-linked spherical protein A clusters of ca. 100 nm at the SPCE surface modified with GA for 120 min (Figure 4c). On the contrary, the electrode modified with protein A without a cross-linking step exhibited a significantly different morphology with fewer and randomly scattered protein A moieties (Figure 4b). Considering the results of this study, further optimization steps were carried out using V_2_CT*_x_*/Nafion SPCEs modified in a 2.5% GA solution for 120 min prior to protein A immobilization.

Before testing the analytical performance, we conducted an optimization study considering the incubation time of the SARS-CoV-2 spike protein. The study was aimed at finding the most intense signal, i.e., the difference between the *R*_ct_ determined from EIS measurements for the sensor incubated at a relatively low spike protein concentration (10^–5^ μg mL^–1^, i.e., 67 fM) in PBS (*R*_inc_) and the *R*_blank_ (Figure 4d). The investigation of the spike protein incubation time showed that 5 min of incubation was insufficient to obtain a signal 3-times higher than the standard deviation of the *R*_blank_, whereas the highest analytical signal was observed after 15 min of incubation. It was observed that for times longer than 15 min, the analytical signal decreased and reached a plateau due to the equilibrium state between the forward and reverse reaction, i.e., binding and dissociation of antigen, respectively. In solution, the antibody–antigen reaction occurs in a few seconds,^43^ and to reach complete equilibrium, where a significant proportion of antibodies are bound to the antigen, more time is required, ranging from minutes to hours. On the other hand, the forward reaction between the antibody and antigen at the solid–liquid interfaces is constrained by both the geometry and organization of the receptor molecules.^44^ This means the surface reactions are less pronounced and take longer to reach equilibrium than those in solution. To tackle this, the incubation solution was applied via a droplet of 10 μL, which concentrated the antigen in the proximity of the antibody-modified electrode, potentially leading to more effective binding and faster equilibrium. However, since the sharp increase of the forward reaction was observed already after 15 min, characterized by a stable charge transfer signal, we incorporated this incubation time for all further tests. Such a short incubation time fulfills the requirements of rapid on-site applications that match the performance of existing so-called rapid commercial (qualitative only) serological and molecular assays.^45^

The stepwise fabrication of the immunosensor described above was monitored via CV and EIS, as shown in Figure S5. The change of the vanadium- and iron-related signals in cyclic voltammograms performed in 1.0 mM [Fe(CN)_6_]^3–/4–^ in 0.1 mol L^–1^ KCl at the V_2_CT*_x_*/Nafion SPCEs was used to follow the sequential addition of sensing layers, i.e., GA as a cross-linker, protein A, anti-SARS-CoV-2 antibody, and BSA as a blocking agent (see Figure S5a). As the electrode becomes covered with mostly negatively charged components, the diffusion of negatively charged redox probes toward available electrode sites decreases. At the same time, the thicker sensing layer decreases the ion exchange efficiency needed for the successful operation of the vanadium redox activity. The successful incorporation of proteins, i.e., protein A, antibodies, and BSA, was confirmed by gradual increases in the size of the Nyquist semicircles corresponding to the charge transfer resistance at the sensor interface, indicating sequential changes in the sensor architecture (Figure S5b).

### 3.3 XPS and ToF-SIMS Studies of the Sensor Architecture

**Figure 5:**
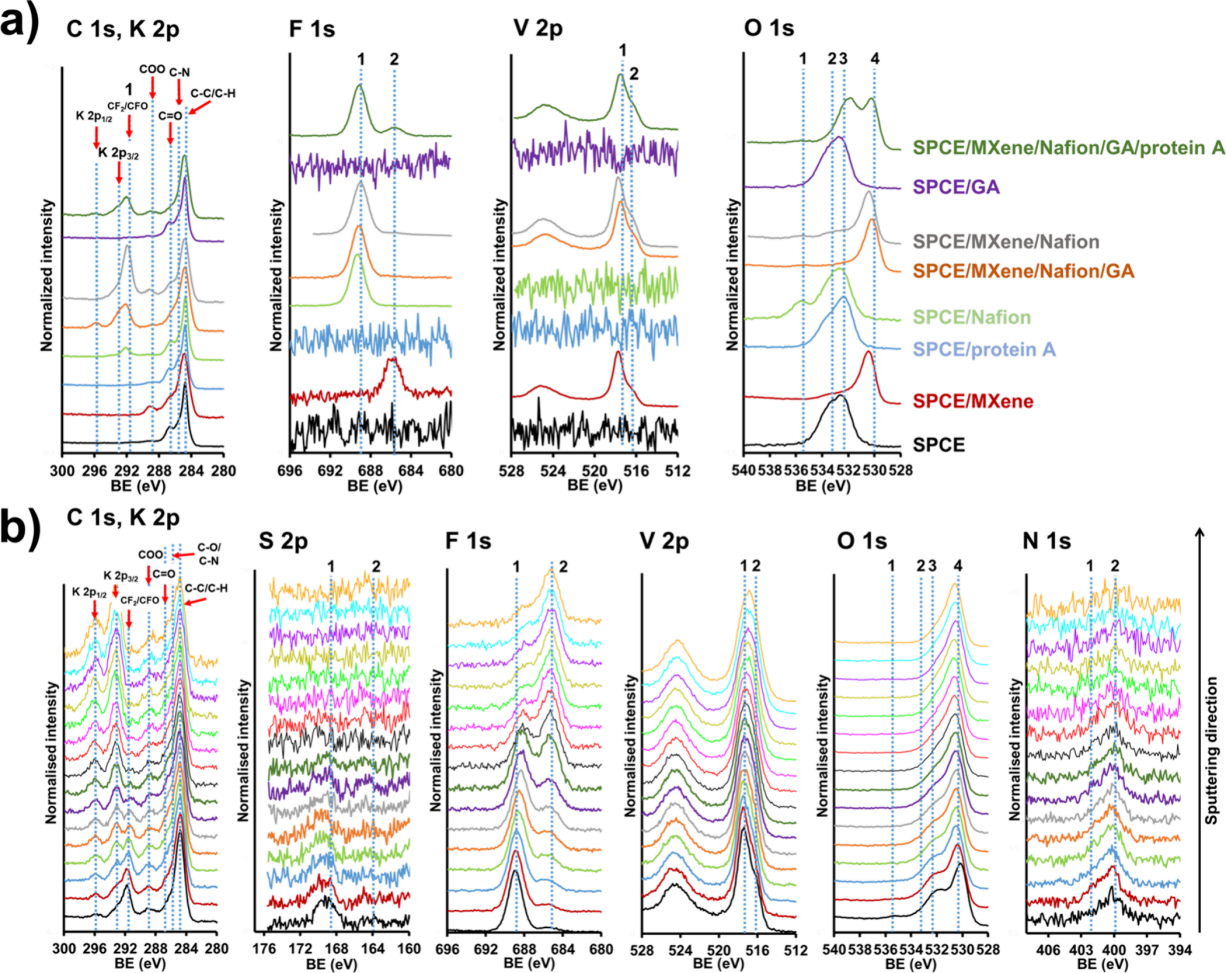
High-resolution XPS spectra (a) of C 1s and K 2p, F 1s, V 2p, and O 1s for different samples, and (b) obtained during depth profiling with the 5 keV Ar_2000_^+^ of C 1s, S 2p, F 1s, V 2p, O 1s, and N 1s for the SPCE/MXene/Nafion/GA/protein A sample. The lowest spectra presented in part b correspond to the measurements before sputtering.

The sensor architecture during the fabrication process was investigated using XPS and ToF-SIMS techniques. Figure 5a shows high-resolution XPS spectra recorded at different fabrication stages. Potassium (K 2p doublet) was detected in some samples but not in all. It may originate from the PBS residues and potassium hexacyanoferrate(II/III) used to characterize the electrochemical behavior of SPCE when obtained and before their modification. The main peak in the C 1s spectra at a BE of 284.8 eV corresponds to the C–C/C–H (present in all samples). The shoulder on the more positive binding energy (BE) side of the main peak represents the C–N signal originating from the amino acids that are the major building blocks of protein A (blue spectrum in Figure 5a). The peak at ca. 292 eV is attributed to the CF_2_/CFO in Nafion. This peak is present for the Nafion-contained samples and decreases as more layers are applied to the Nafion, i.e., GA and protein A (see orange and dark green spectra in Figure 5a).^46^ On the other hand, MXene-related features corresponding to C=O and O–C=O are located at ca. 287 and 289 eV, respectively.^47^ These features are present in all samples containing V_2_CT_*x*_ MXenes. The F 1s spectra confirm the presence of Nafion and MXene on the electrode surface. The position at the dashed line 1 at ca. 689 eV corresponds to the position of the Nafion-based fluorine signal,^48^ while the fluorine terminal groups in MXene are described by a low-intensity peak at more negative BE (at the position of dashed line 2, approximately at 685 eV).^49^ As for the vanadium features, a clear doublet of the V 2p spectra is observed. The position of the V 2p_3/2_ indicates that vanadium is present in the V(II), V(III), and V(IV).^47,50,51^ In addition, the position at the dashed line 1 at ca. 517 eV corresponds to V–O, while the low-intensity shoulder at ca. 516 eV (line 2) can be associated with V–C. Since oxygen is present in all components, the corresponding O 1s spectra provide deeper information about the architecture of our sensor and its stepwise fabrication. Bare SPCE contains two features at ca. 532 and 534 eV, both of which are due to oxygen originating from organic molecules or bound water molecules on the SPCE surface (positions at dashed lines 2 and 3). On the other hand, the signal from oxygen belonging to the sulfonate groups in Nafion develops at ca. 535 eV (dashed line 1), which was observed in the SPCE/Nafion sample and other samples containing Nafion. In the SPCE/MXene sample, the feature at the most negative BE at ca. 530 eV (dashed line 4) belongs to O in V–C–O and/or V–C–OH originating from MXene.^49^ In the case of protein A adsorbed on the surface of SPCE, i.e., for the sample SPCE/protein A (O 1s blue spectrum in Figure 5a), the O 1s spectrum is slightly different from that of the bare SPCE, likely due to the contribution of the amino acids, i.e., C=O, C–O, and O–C=O with BE positions at ca. 532 eV. When the SPCE is modified with both MXene and Nafion (O 1s gray spectrum in Figure 5a), the spectrum is dominated by the features belonging to the oxygen in MXene (the same as for red spectrum in Figure 5a), while the oxygen from the sulfonate groups of Nafion from SPCE/MXene/Nafion is present at the same position (O 1s gray spectrum in Figure 5a) as in the SPCE/Nafion sample (O 1s green spectrum in Figure 5a, position at dashed line 1). Finally, an intense feature appears at ca. 532 eV after the addition of protein A, which can be assigned to its oxygen belonging to the hydroxyl, carbonyl, or carboxyl groups of amino acids.

Additional XPS spectra are presented in the Supporting Information. The peak in Al 2p spectra was detected for the sample SPCE/MXene/Nafion/GA/protein A (Figure S6a). As shown above, in the discussion concerning the EDX analysis, about 1 wt % Al is randomly distributed on the surface of the electrode and belongs to the parental MAX phase and/or aluminum (oxo)fluoride particles; the contribution of aluminum in the XPS spectra is incidental with respect to the acquisition site and was observed only in the complete architecture of SPCE/MXene/Nafion/GA/protein A.

As the Al 2p peak was present, it suggests that the remaining sparse Al moieties on the surface are exposed as inactive areas, implying that they do not interact with additional functional layers. Additionally, Cl 2p and Si 2p spectra shown in Figures S6b and S6c belong to SPCEs originating from binders commonly used for the preparation of screen-printing carbon pastes. Therefore, the signals of these two elements were only detected in samples not covered with MXene, where the coating was thinner than the sampling depth of the XPS technique (ca. 5–10 nm), i.e., at the SPCE/GA, SPCE/Nafion, and SPCE/protein A. As expected, the complete sensor architecture has no Cl 2p and Si 2p signals. The N 1s spectra are shown in Figure S6d. The nitrogen signal mainly comes from the amino groups, with a characteristic feature at about 400 eV, which is most intense for adsorbed protein A in sample SPCE/protein A (blue spectrum in Figure S6d) and in chemically bound protein A to GA (dark green spectrum in Figure S6d). It is also observable in the sample SPCE/MXene at the position of 402 eV (red spectrum in Figure S6d), corresponding to the binder of the screen-printing paste, or hexacyanoferrate from SPCE pretests explained above in the manuscript.

In addition to the XPS analyses for each stage of the sensor fabrication process, Figure 5b demonstrates spectra obtained during depth profiling using a 5 keV Ar_2000_^+^ for the SPCE/MXene/Nafion/GA/protein A sample. The lowest spectra represent the surface of the sample before sputtering, whereas the upper spectra are stacked based on the sputtering progress through the sample. In the C 1s and K 2p spectra, potassium signal was observed, originating from the potassium hexacyanoferrate(II/III) residues during SPCE characterization. The signal K 2p increases with sputtering and has a more significant contribution closer to the surface of the SPCE, as can be seen in the upper spectra (K 2p spectra in Figure 5b). Also, increasing the sputtering time decreases the CF_2_/CFO signal as the Nafion is gradually removed from the sensor surface. In the S 2p spectra, the signal has low intensity and is more pronounced closer to the SPCE surface, which can be attributed to the Nafion layer. The analyzed spot size for XPS measurements during depth profiling is smaller, i.e., the diameter of the analyzed area during depth profiling was 110 μm, whereas for the spectra acquisition shown in Figure 5a, a larger area was analyzed, i.e., 300 by 700 μm. Thus, the intensity of species with low surface atomic concentrations is less pronounced during depth profiling. This is most likely why the S 2p signal corresponding to protein A, i.e., from methionine amino acids, was not developed (positioned at dashed line 2). However, the N 1s signal originating from protein A is present (described below). The F 1s spectra shown in Figure 5b reveal a BE shift in the signal representing Nafion (dashed line 1) to the underlying MXene (dashed line 2) as sputtering progresses. The environment of V does not change significantly during sputtering, as seen in the V 2p spectra in Figure 5b; however, it is noticeable that the low BE side of the most intense peak is more pronounced for spectra representing regions closer to the electrode surface, suggesting that the upper part of the MXene layer consists of higher valence V(IV) states. As sputtering progresses, the peak corresponding to O in inorganic species, i.e., V–C–O bond, becomes more intense (position on dashed line 4), and the high BE features of O in organic species associated with protein A become less intense with sputtering (positions at dashed lines 1–3), as the organic species are gradually removed during the sputtering process. Finally, as expected, the signal for N, originating from protein A (in particular from amino groups), is more intense at the topmost position (lower N 1s spectra) and decreases during the sputtering procedure. The depth profiles (surface atomic concentration vs sputtering time) of the main species for the SPCE/MXene/Nafion/GA/protein A sample are shown in Figure S7.

**Figure 6:**
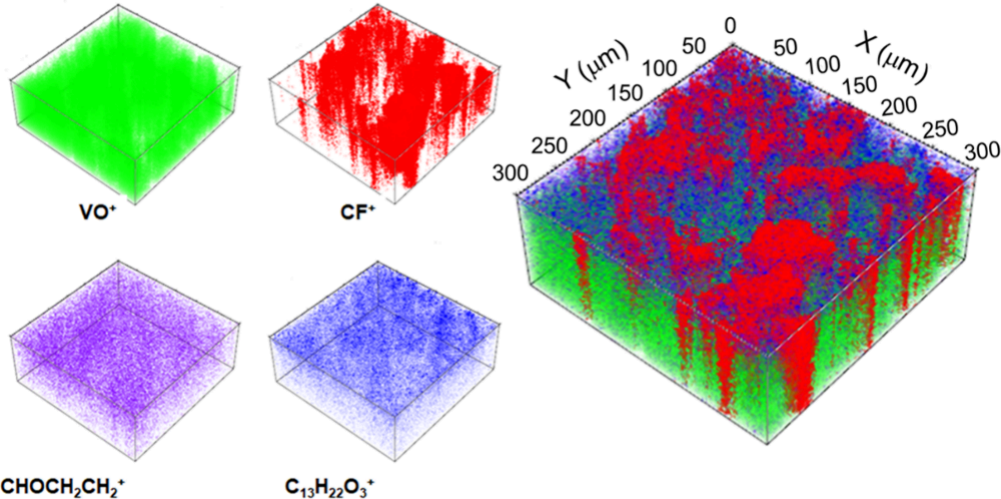
3D ToF-SIMS images showing the distribution of main constituents of sensor architecture. The signal representing V_2_CT_*x*_ MXene is shown in green, Nafion in red, GA in violet, and protein A in blue.

The ToF-SIMS technique was used to determine the spatial distribution of the constituents within our sensor architecture before introducing the antibody. 3D ToF-SIMS profiles were measured in positive (Figure 6) and negative (Figure S8) polarity. VO^+^ or VO_2_^–^ signals were used to represent V_2_CT_*x*_ MXene; CF^+^ and CF_3_^–^ signals were employed to determine the spatial distribution of Nafion (C_7_HF_13_O_5_S·C_2_F_4_); CHOCH_2_CH_2_^+^ signal represents a fragment ion from GA (C_5_H_8_O_2_); protein A was characterized by C_13_H_22_O_3_^+^ and C_3_HO_3_^–^ fragments. The corresponding MXene signals, i.e., both positive (VO^+^) in Figure 6 and negative (VO_2_^–^) in Figure S8, are evenly distributed in the observed region, which is in agreement with the presented 3D profilometry, microscopy analysis, and the elemental XPS depth profiling. The Nafion membrane, which serves to immobilize the particles, appears to be evenly distributed when the contributions of the negative CF^–^ moieties are taken into account (Figure S8). The contribution of GA is clearly visible and is based on the coherent CHOCH_2_CH_2_^+^ signal; moreover, GA forms visibly homogeneous cross-linking support for the upstream protein A molecules. The latter are characterized by a signal deriving from high carbon, hydrogen, and oxygen moiety C_13_H_22_O_3_^+^. Similar moieties have already been used for protein identification by the means of ToF-SIMS.^52^ The observed distribution agrees well with the results of the FE-SEM analysis, which showed protein A clusters on the top of the modified electrode that typically form in the presence of the GA cross-linker.

### 3.4 Analytical Performance of the Sensor

**Figure 7:**
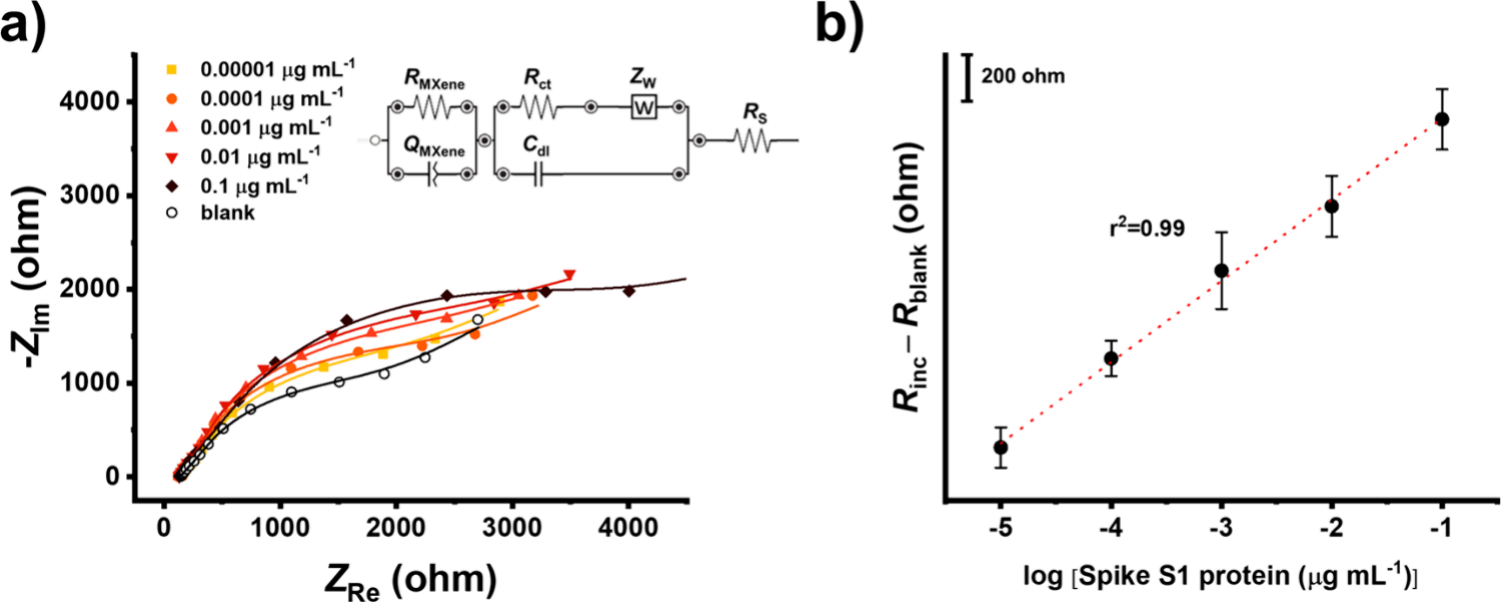
(a) EIS response for increasing concentrations of spike protein in PBS (pH = 7.4, six replicates) and (b) the corresponding calibration curve of the fully optimized sensor. The obtained spectra were fitted using the equivalent circuit shown in the inset of part a and explained in Methods.

Due to the well-known intrinsic irreproducibility, the SPCEs were first subjected to the square-wave voltammetric characterization in 1.0 mmol L^–1^ [Fe(CN)_6_]^3–/4–^ in 0.1 mol L^–1^ KCl, and only those with the peak current falling into the range of 50–60 μA were used for further modifications to prepare immunosensors; this voltammetric technique offers improved sensitivity versus cyclic voltammetry, particularly for electrochemically reversible redox systems. The electroanalytical performance of the V_2_CT_*x*_ MXene-based immunosensor was investigated by increasing the concentration of spike protein in the broad range of 10^–5^ μg mL^–1^ – 0.1 μg mL^–1^ (Figures 7a and 7b). The immunosensor revealed a linear *R*_inc_ – *R*_blank_ response (*r*^2^ = 0.99) in the examined concentration range in combination with a short incubation time of only 15 min. Using the 3σ criteria, a very low limit of detection of 45 fM was obtained. In addition, the intraday reproducibility calculated as the relative standard deviations of 8% and 9% were observed for nonincubated sensors and sensors incubated with 10^–5^ μg mL^–1^ of spike protein, respectively.

**Figure 8:**
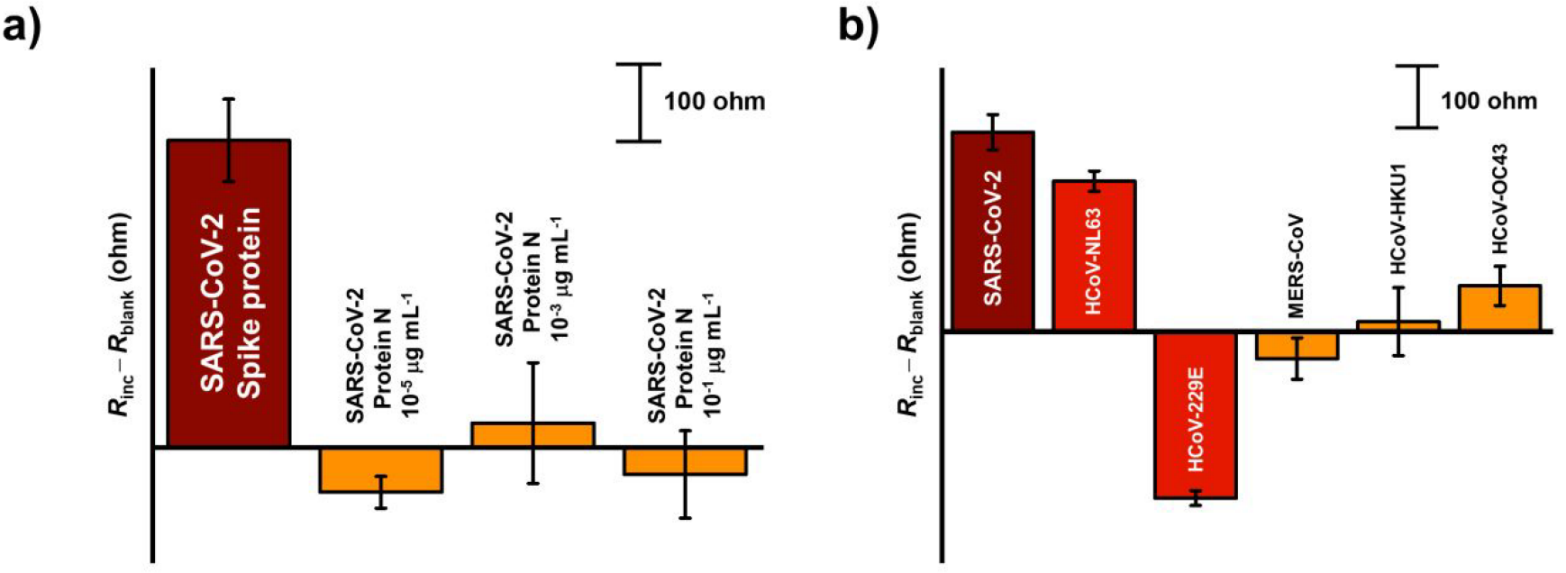
(a) EIS signals for three different concentrations of protein N and 10^–5^ μg mL^–1^ spike protein (pH = 7.4, three replicates) and (b) EIS signals obtained for equal concentration (10^–3^ μg mL^–1^) of different coronaviruses, i.e., SARS-CoV-2, HCoV-NL63, HCoV-229E, MERS-CoV, HCoV-HKU1, and HCoV-OC43.

We tested the immunosensor in the presence of a model interferent SARS-CoV-2 nucleocapsid protein, also known as protein N, at three different concentrations, i.e., 10^–5^, 10^–3^, and 10^–1^ μg mL^–1^. Surprisingly, negligible changes in the *R*_ct_ were observed upon incubation for all three investigated protein N concentrations (Figure 8a). The *R*_ct_ values were in the range of a blank signal and significantly lower compared to the signal obtained for a low concentration of the target spike protein, indicating a favorable selectivity of the newly developed immunosensor.

To thoroughly investigate the potential of our sensor, we also performed a selectivity study including spike proteins of several human coronaviruses of similar structures to SARS-CoV-2, i.e., HCoV-OC43, HCoV-HKU1, HCoV-NL63, HCoV-229E, and MERS-CoV. All of these viruses commonly cause mild to moderate upper respiratory tract illnesses in humans. Their symptoms can overlap with the symptoms caused by SARS-CoV-2. The results of this study are presented in Figure 8b. It can be noted that MERS-CoV, HCoV-HKU1, and HCoV-OC43 did not cause any significant interference, with observed selectivity factors of −13%, +5%, and +23%, respectively. The selectivity factors were calculated as a ratio between the EIS signal of interferent and SARS-CoV-2. On the other hand, HCoV-NL63 showed a somewhat higher signal contribution with a selectivity factor of +75%. Interestingly, a substantial decrease in the analytical signal occurred upon incubation with HCoV-229E with a selectivity factor of −81%. Considering the latter results, it is rather challenging to completely interpret the observed phenomenon; the higher molecular weight of the HCoV-229E spike protein compared to other investigated candidates, as well as its positive net charge, may explain the unexpectedly low signal. On the contrary, structural similarities to SARS-CoV-2 are notably the most pronounced in the case of HCoV-NL63, resulting in the highest positive selectivity factor observed.

Finally, we carried out measurements in the artificial nasopharyngeal fluid (ANF) spiked with 100 μg mL^–1^ spike protein. Nasopharyngeal fluid, being rich in diverse components, such as mucus, proteins, protein mimics, salts, ions, and cells, can strongly influence the immunosensor’s operation. Thus, the spiked nasopharyngeal samples present a big challenge for the electrochemical immunosensing platforms. To attenuate the matrix effect of the nasopharyngeal fluid, we implemented dilutions of the sample with PBS (pH = 7.4), i.e., 10, 20, and 50 times. A linear signal for three consecutive dilutions (*r*^2^ = 0.99, *n* = 3) was obtained, along with a sensitivity of 51.22 ± 6.26 Ω·mL·μg^–1^, as shown in Figure S9a,b. Notably, likely due to the matrix effect, the shape of the obtained EIS spectra was affected so that the contribution from the MXene-modification layer, characterized by determined *R*_ct_ when incubated with the spike protein samples in PBS, was not observed and was excluded from the corresponding equivalent circuit; this phenomenon is attributed to the sluggish ion diffusion. Thus, to process the obtained data, an even simpler Randles-type circuit was used, i.e., *R*_S_([*R*_ct_*Z*_W_]*Q*_dl_). The obtained values for the analytical signal, i.e., *R*_inc_ – *R*_blank_, were lower in comparison to the values obtained for the samples in PBS, accounting for the idle kinetics of the antigen–antibody reactions in a complex matrix within the incubation time frame of 15 min. Under the given conditions, the reactivity of SARS-CoV-2 spike protein toward antibodies immobilized on the surface can be reduced due to interactions with other glycoproteins or entrapment by macrophages and dendritic cells of ANF. Although the accuracy of the measurements was slightly compromised, the sensor exhibited high linearity and good sensitivity, demonstrating its potential for reliable detection of SARS-CoV-2 spike protein. Furthermore, due to potential pH fluctuations in real samples, the operation of the immunosensor was tested in samples with different pH values, i.e., 5, 6, 7.5, and 8. Interestingly, the experiments revealed the highest response (*R*_inc_ – *R*_blank_) in the sample with pH 7.5, and the samples with pH 5 and 6 showed almost the same responses which were ca. 25% lower than that at pH 7.5, whereas the measurement in the sample with pH 8 exhibited a signal ca. 14% lower compared to the signal recorded at pH 7.5 (Figure S10).

Such electroanalytical performances can be attributed to favorable interlayer synergies of the presented immunosensor’s architecture. This includes, apart from the inherent sensitivity of the EIS technique, a stable and (electro)catalytically active layer of V_2_CT*_x_* MXene flakes immobilized in Nafion film, which simultaneously serves as an effective support for glutaraldehyde deposition, the latter enabling an efficient binding of protein A for further site-oriented covalent immobilization of anti-SARS-CoV-2 antibodies. At the same time, the application of BSA as a blocking agent against unspecific binding events still allowed a very sensitive functioning of the immunosensor. In comparison with other similar impedimetric immunosensors for the SARS-CoV-2 antigen listed in Table S2, we can conclude that our immunosensor compares favorably or even surpasses them, considering the overall sensing performances.^53−61^

## 4 Conclusions

An immunosensor based on V_2_CT*_x_* MXene exhibited excellent sensitivity and satisfactory selectivity toward SARS-CoV-2 spike protein in combination with electrochemical impedance spectroscopy. The electrocatalytic MXene was immobilized on the supporting SPCE surface using a thin Nafion layer additionally functionalized with the GA-cross-linked protein A. The optimized immunosensor architecture showed a favorable electrocatalytic capability of V_2_CT*_x_* MXene, enabling sensitive, label-free, and rapid impedimetric detection of the SARS-CoV-2 spike protein with a detection limit as low as 45 fM, in combination with only 15 min incubation. The practical applicability of the immunosensor was demonstrated through the successful detection of SARS-CoV-2 spike protein in artificial nasopharyngeal fluid. Its electroanalytical performance indicates a great potential of the MXene-based interfaces for their broader immunosensing applications and suggests another direction toward developing rapid and point-of-need immunoassays.

## Supporting Information Available

3D profilometry analysis of coatings with different V_2_CT_*x*_ MXene loadings. FE-SEM images of V_2_CT_*x*_ MXene deposited on the SPCE. FE-SEM/EDX analysis of the as-synthesized V_2_CT_*x*_ MXene and elemental composition. Bright-field mode STEM/EDX analysis of the V_2_CT_*x*_ MXene flake with EDX layered image. Step-by-step fabrication of the immunosensor monitored by EIS and CV. High-resolution XPS spectra of different samples. XPS depth profiles for different elements. 3D negative ion ToF-SIMS images of sensing architecture. The calibration curve of the fully optimized immunosensor in ANF. The effect of pH of the ANF on the signal of immunosensor. Comparison of impedimetric immunosensors for the detection of SARS-CoV-2 spike protein (PDF)
