# Supplementary material for: Study of V2CTx-MXene Based Immunosensor for Sensitive Label-Free Impedimetric Detection of SARS-CoV-2 Spike Protein
Source: ACS Appl Mater Interfaces. 2024 May 30;16(23):30196–208. doi: 10.1021/acsami.4c04567 (PMC11181268; doi:10.1021/acsami.4c04567)
Supplement: Supplementary file 1 — am4c04567_si_001.pdf [file am4c04567_si_001.pdf]

Supporting Information

Study of V_2CT_X-MXene Based Immunosensor for Sensitive Label-Free Impedimetric Detection of SARS-CoV-2 Spike Protein

Nikola Tasić,[1] Ivan Konjević,[1,2] Alnilan Lobato,[1,3] Dino Metarapi,[1] Matjaž Finšgar,[4] Filipa M. Oliveira,[5] Zděnek Sofer,[5] Rui Gusmão,[5,*] Xueji Zhang,[6] Samo B. Hočevar[1,*]

[1]Department of Analytical Chemistry, National Institute of Chemistry, Hajdrihova ulica 19, 1000 Ljubljana, Slovenia

[2]Faculty of Chemistry and Chemical Technology, University of Ljubljana, Večna pot 113, 1000 Ljubljana, Slovenia

[3]International Postgraduate School Jožef Štefan, Jamova 39, 1000 Ljubljana, Slovenia

[4]Faculty of Chemistry and Chemical Engineering, University of Maribor, Smetanova ulica 17, 2000 Maribor, Slovenia

[5]Department of Inorganic Chemistry, University of Chemistry and Technology Prague, Technická 5, 166 28 Praha 6-Dejvice, Czech Republic

[6]School of Biomedical Engineering, Shenzhen University Health Science Center, 3688 Nanhai Road, Nanshan District, Shenzhen 518054, Guangdong, P.R. China

Corresponding Authors:

[*]Samo B. Hočevar (samo.hocevar@ki.si)

[*]Rui Gusmão (rui.gusmao@vscht.cz)

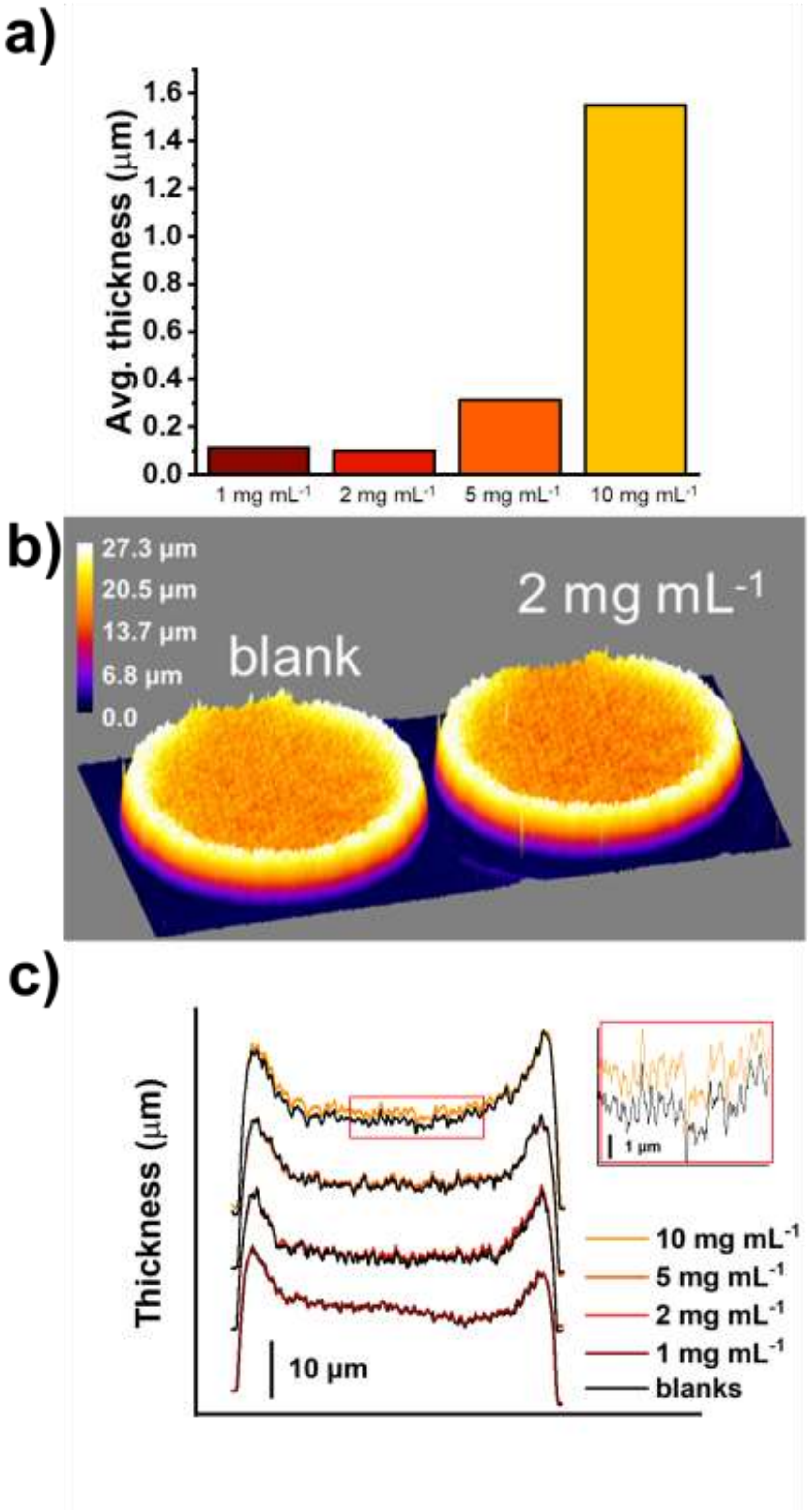

Figure S1. (a) Average thickness of the coatings using different concentrations of V_2CT_x MXene for SPCE modification, (b) 3D profiles of bare SPCE and SPCE modified with 2 mg mL^{-1} V_2CT_x MXene + 0.0025% Nafion, and (c) line sections of bare SPCE and SPCEs modified with different concentrations of V_2CT_x MXene.

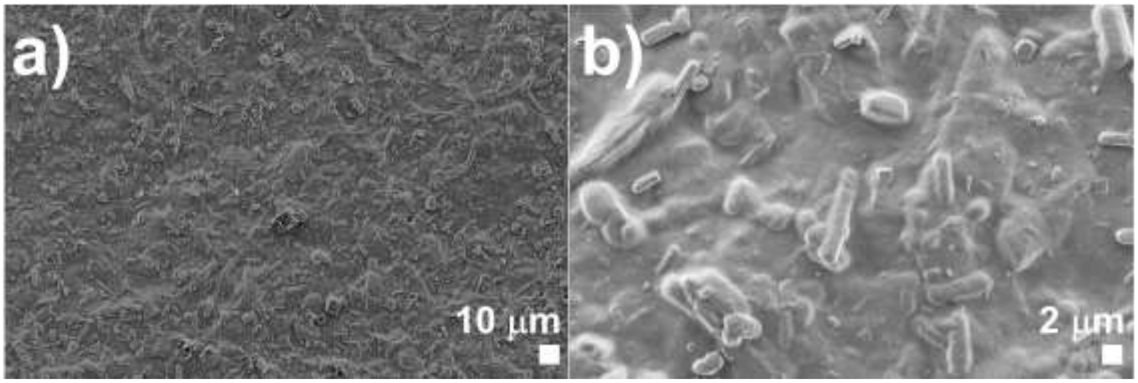

Figure S2. (a), (b) FE-SEM images of V_2CT_x MXene on SPCE after sonication of the drop-casting solution for 15 minutes at 22-30 °C. Both images correspond to the same sample recorded using different magnifications.

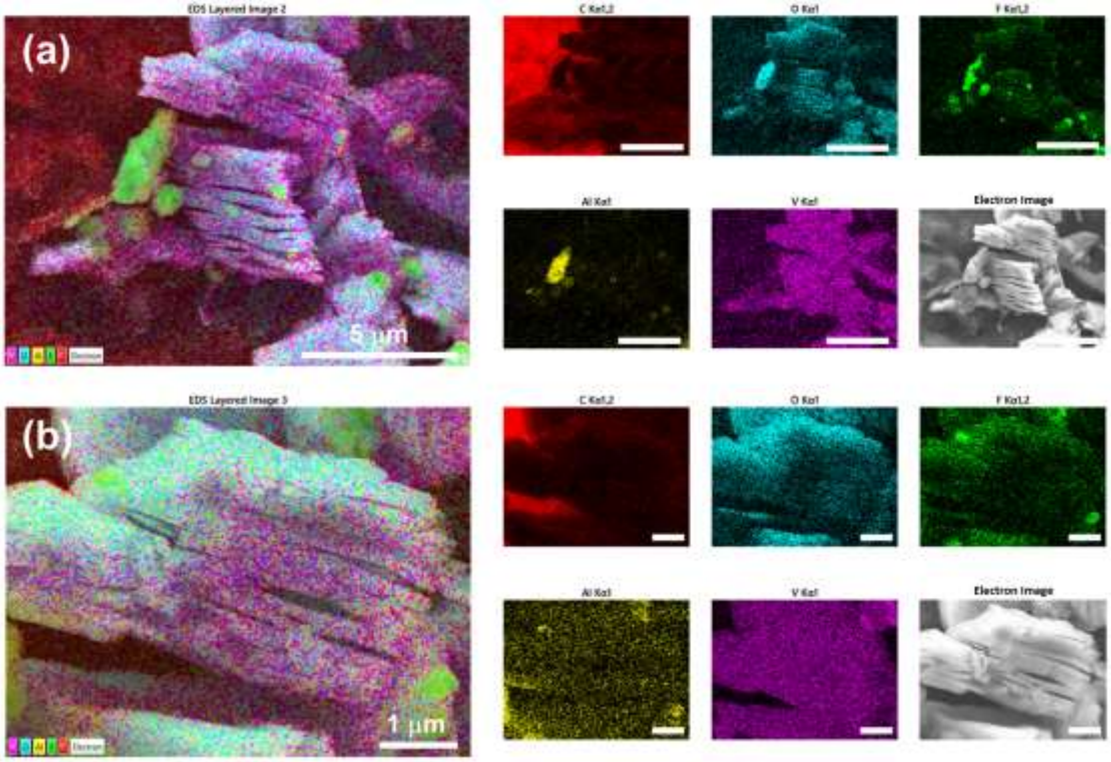

Figure S3. FE-SEM/EDX analysis of the as-synthesized V_2CT_x MXene drop-casted on SPCE (a, b). Scale bars in the images on the right are identical to those on the left.

Table S1. The elemental composition (wt.%) of samples presented in **Figure S3**.

Element	C	V	O	F	Al	Cl	S
Upper site on SPCE (a)	52.2	31.8	8.3	5.6	1.4	0.6	0.2
Magnified site on SPCE (b)	56.7	24.6	10.8	6.6	0.7	0.5	/

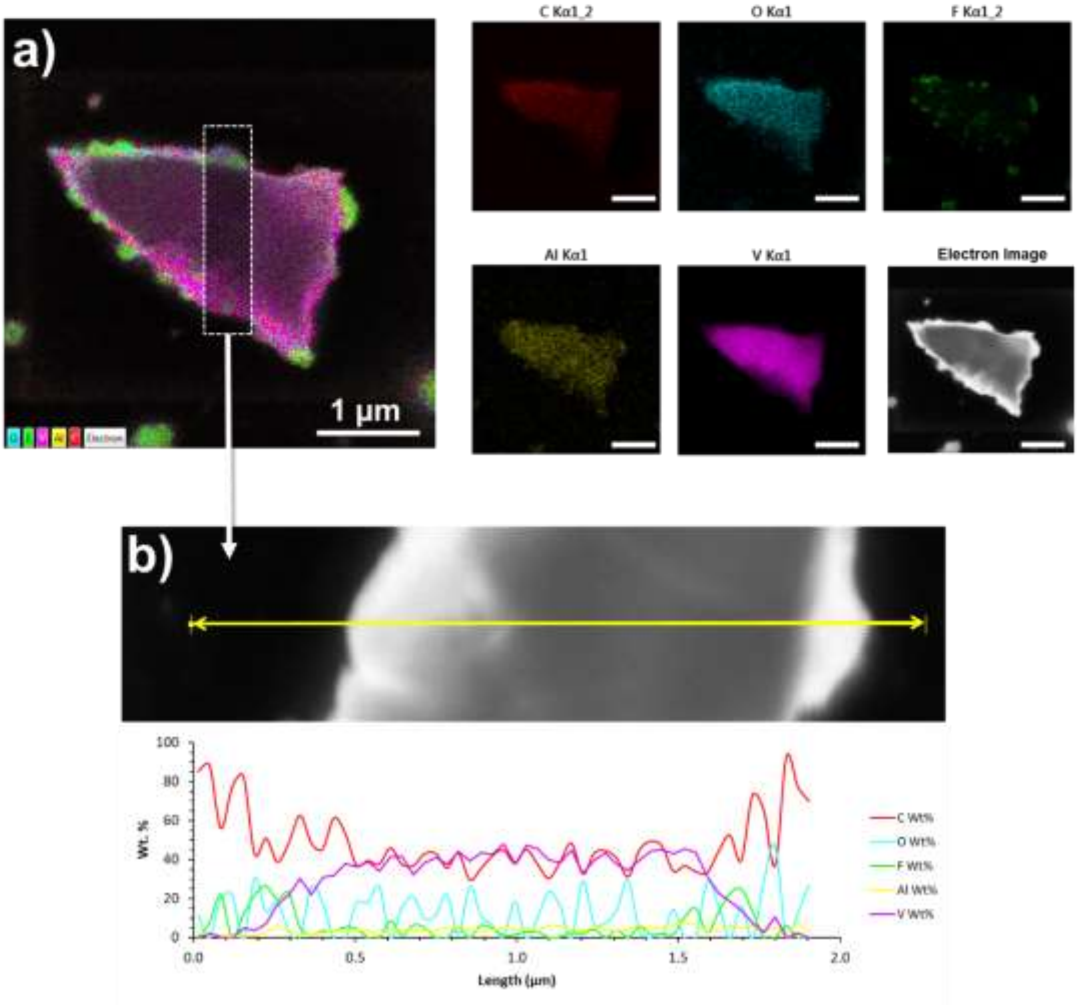

Figure S4. (a) Bright-field mode STEM/EDX analysis of the V_2CT_x MXene flake with EDX layered image, respective map of elements and electron micrograph, (b) detail of the area used for EDX 1D line scan analysis of the V_2CT_x MXene flake and respective profile of detected elements (wt. %). The scale bar represents 1 µm, and the length of the line scan is ca. 1.9 µm.

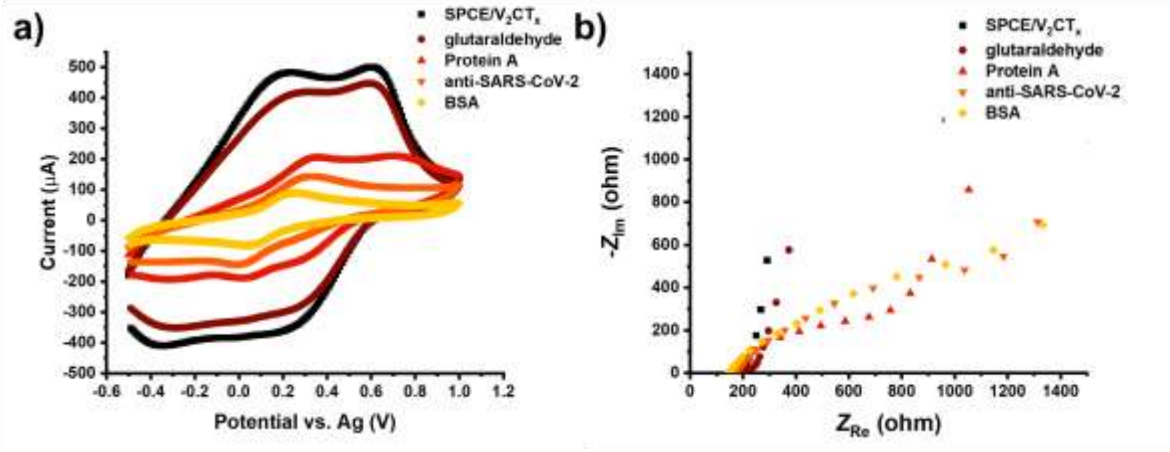

Figure S5. Step-by-step fabrication of the immunosensor followed by (a) cyclic voltammetry and (b) EIS (Nyquist spectra) in the presence of 1.0 mmol L^{-1} $[Fe(CN)_6]^{3-/4-}$ in 0.1 mol L^{-1} KCl.

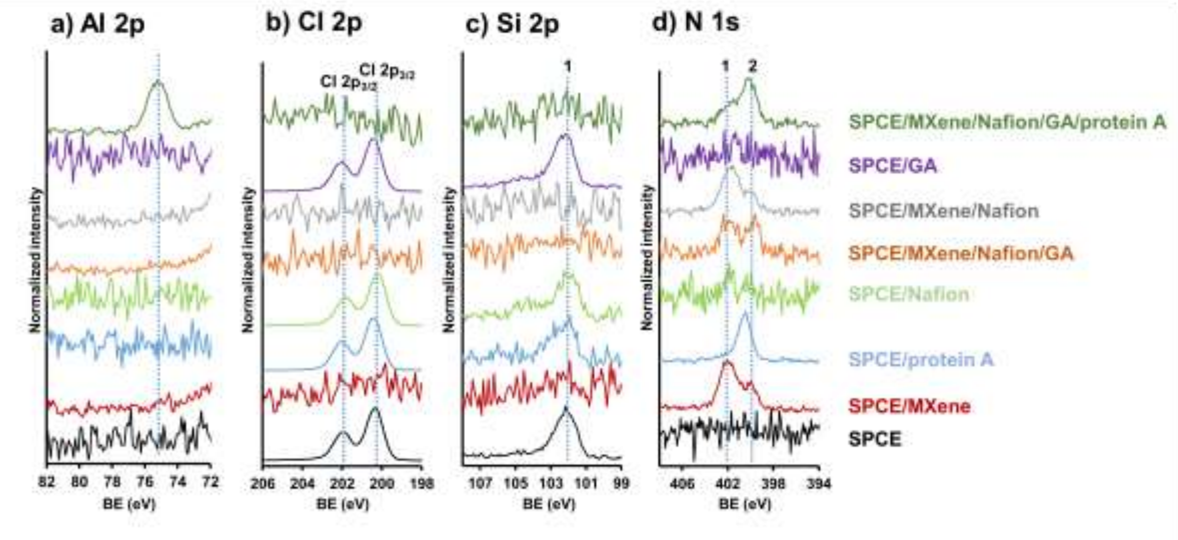

Figure S6. High-resolution XPS spectra of (a) Al 2p, (b) Cl 2p, (c) Si 2p, and (d) N 1s for different samples.

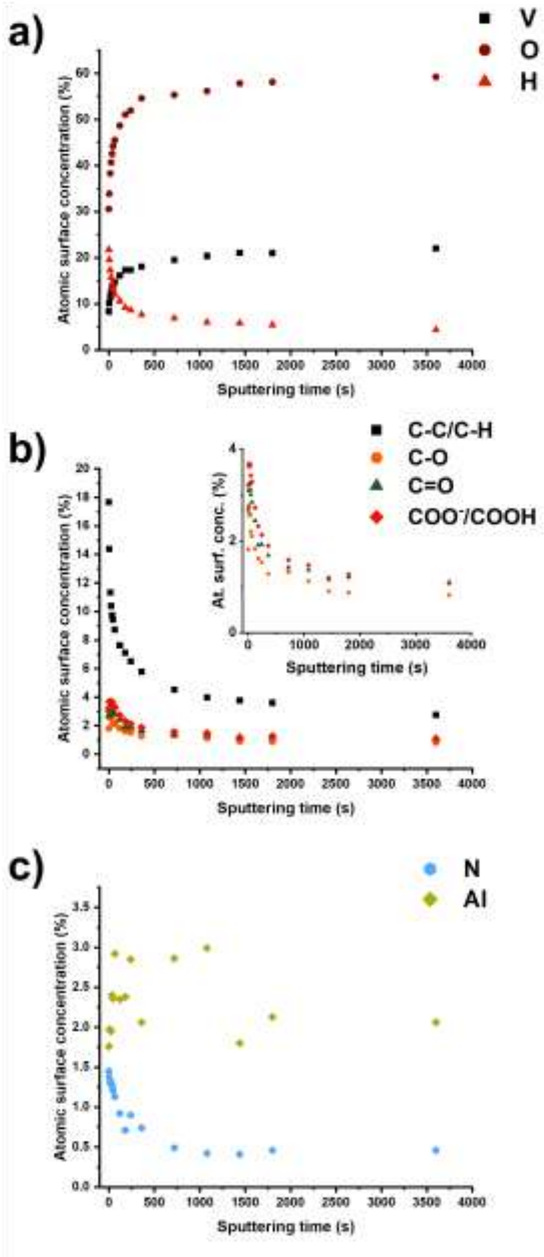

Figure S7. XPS depth profiles showing (a) V, O, F, (b) C-based compounds, and (c) N and Al.

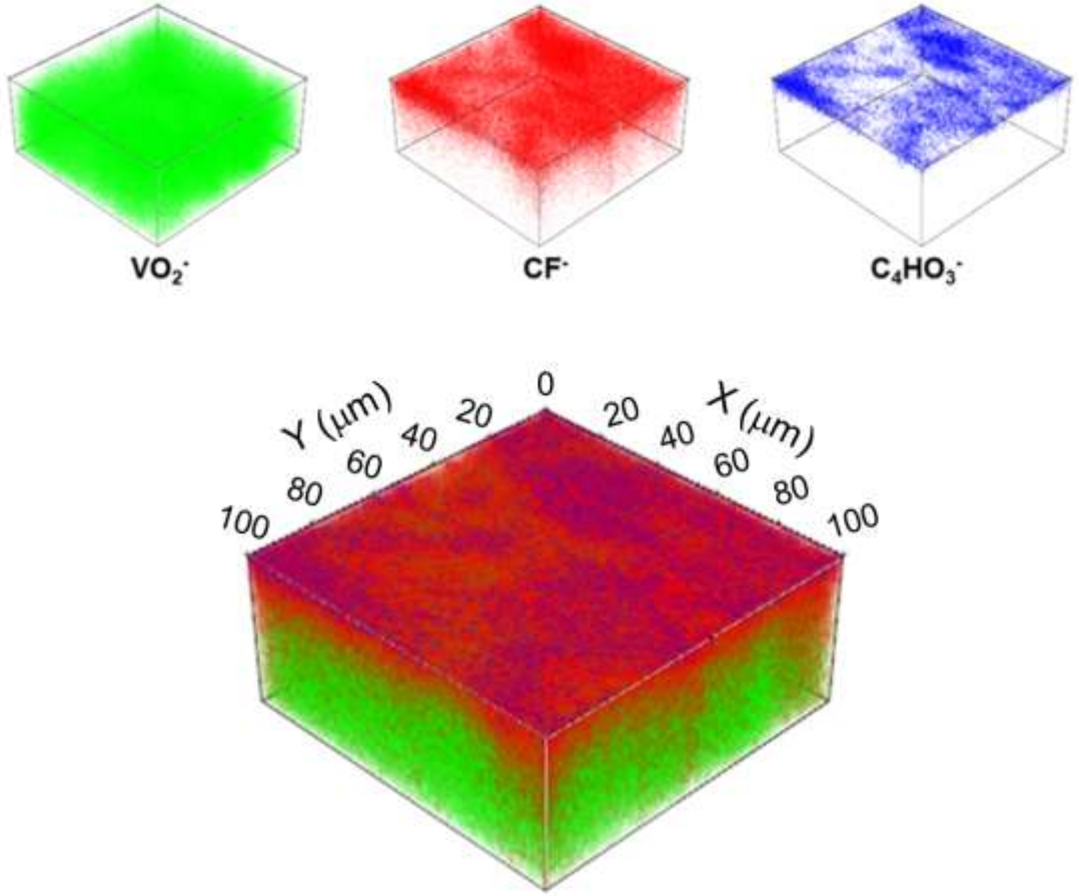

Figure S8. 3D negative ion ToF-SIMS images depicting the distribution of main constituents of our sensing architecture.

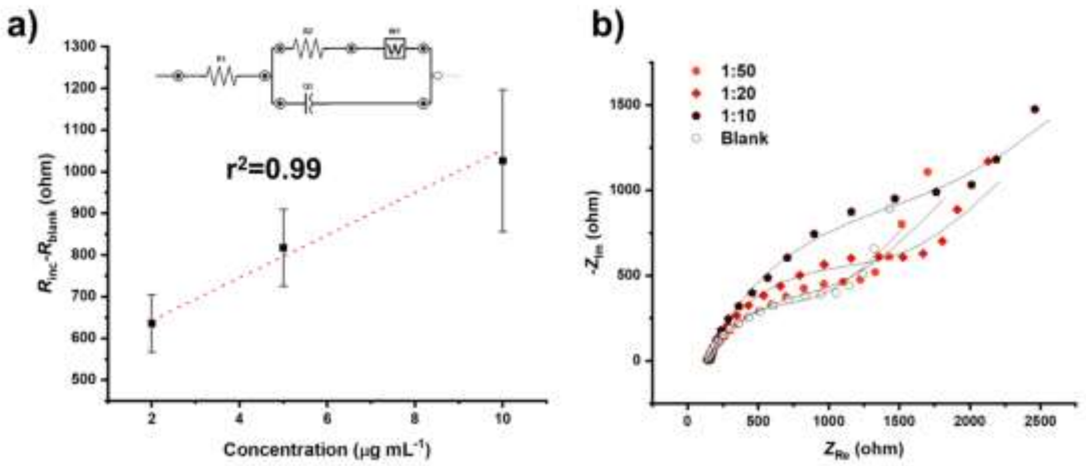

Figure S9. (a) Calibration curve of the fully optimized sensor, obtained in the diluted SARS-CoV-2 Spike protein sample in artificial nasopharyngeal fluid. The concentration of the sample was 100 μg mL^{-1}, and the tested dilutions of 1:10, 1:20, and 1:50 correspond to concentrations of 10, 5, and 2 μg mL^{-1}, respectively. The obtained spectra were fitted using the equivalent circuit shown in the inset of **Figure S9a,** (b) Corresponding Nyquist spectra for different sample dilutions.

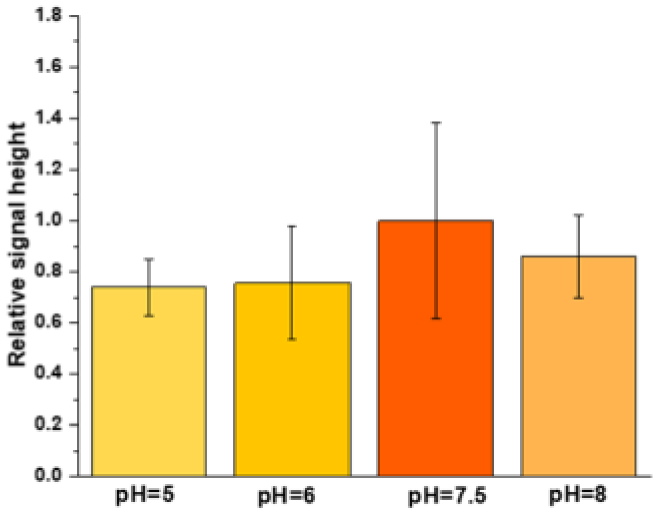

Figure S10. The effect of pH of the ANF on the signal of immunosensor.

Table S2. Comparison of the existing impedimetric immunosensors for the detection of SARS-CoV-2 Spike protein.

Sensing base	Range	LOD	Ref.
SPCE/ SiO_2 UiO-66 (MOF)	100 fg mL^{-1} to 10 ng mL^{-1}	100 fg mL^{-1} (1.27 fM)*	53
Au electrode	0.05 to 10 µg mL^{-1}	as low as 0.1 µg mL^{-1} (1.28 nM)*	54
SPE/MWCNT	0.05 to 1 µg mL^{-1}	20±1.8 ng mL^{-1} (0.255 nM)*	55
SPCE/gelatin	0.001 to 10 µg mL^{-1}	90 pg mL^{-1} (1.15 pM)*	56
AuSPE	700 ng mL^{-1} to 7 µg mL^{-1}	as low as 38.99 ng mL^{-1} (0.498 nM)*	57
SPCE/Cu_2O nanocubes	0.25 fg mL^{-1} to 1 µg mL^{-1}	0.04 fg mL^{-1} (0.51 zM)*	58
SPCE/electrodeposited Au NPs	10^{-7} to 10^{-11} mol L^{-1}	3.16 pM	59
AuSPE	11.56 to 740 ng mL^{-1}	5.65 pg mL^{-1} (72.2 fM)*	60
AuIDE	250 to 8000 fM	189 fM	61
SPCE/ V_2CT_x	10^{-5} to 10^{-1} µg mL^{-1}	45 fM	this study

SPCE – screen printed carbon electrode, MOF – metal organic framework, SPE – screen printed electrode, AuIDE – gold interdigitated electrode. *Molar concentrations were calculated from the reported mass concentrations, by using the molecular weight for Spike S1 protein of 78.3 kDa.
